# Analysis of chicken macrophage functions and gene expressions following infectious bronchitis virus M41 infection

**DOI:** 10.1186/s13567-021-00896-z

**Published:** 2021-01-28

**Authors:** Xiaoqi Sun, Zheng Wang, Changhao Shao, Jia Yu, Haoyun Liu, Huijie Chen, Lu Li, Xiurong Wang, Yudong Ren, Xiaodan Huang, Ruili Zhang, Guangxing Li

**Affiliations:** 1 Department of Basic Veterinary Science, College of Veterinary Medicine, Heilongjiang Key Laboratory for Animal and Comparative Medicine, Northeast Agricultural University, Harbin, 150030 China; 2 Large Scale Instrument and Equipment Sharing Service Platform, Northeast Agricultural University, Harbin, 150030 China; 3 State Key Laboratory of Veterinary Biotechnology, Harbin Veterinary Research Institute, Chinese Academy of Agricultural Science, Harbin, 150069 China; 4 Department of Computer Science and Technology, College of Electrical and Information Technology, Northeast Agricultural University, Harbin, 150030 China

## Abstract

Infectious bronchitis virus (IBV) is a pathogenic coronavirus with high morbidity and mortality in chicken breeding. Macrophages with normal biofunctions are essential for host immune responses. In this study, the HD11 chicken macrophage cell line and chicken peripheral blood mononuclear cell-derived macrophages (PBMCs-Mφ) were infected with IBV at multiplicity of infection (MOI) of 10. The dynamic changes of their biofunctions, including cell viability, pathogen elimination function, phagocytic ability, and gene expressions of related proteins/mediators in innate and acquired immunity, inflammation, autophagy and apoptosis were analyzed. Results showed that IBV infection decreased chicken macrophage viability and phagocytic ability, and increased pathogen elimination function. Moreover, IBV augmented the gene expressions of most related proteins in macrophages involved in multiple host bioprocesses, and the dynamic changes of gene expressions had a close relationship with virus replication. Among them, MHCII, Fc receptor, TLR3, IFN-α, CCL4, MIF, IL-1β, IL-6, and iNOS showed significantly higher expressions in IBV-infected cells. However, TLR7, MyD88, MDA5, IFN-γ, MHCII, Fc receptor, MARCO, CD36, MIF, XCL1, CXCL12, TNF-α, iNOS, and IL-10 showed early decreased expressions. Overall, chicken macrophages play an important role in host innate and acquired immune responses to resist IBV infection, despite early damage or suppression. Moreover, the IBV-induced autophagy and apoptosis might participate in the virus-host cell interaction which is attributed to the biological process.

## Introduction

Infectious bronchitis (IB) is a highly contagious respiratory disease in chickens with worldwide distribution and economic significance [1]. IB is caused by infectious bronchitis virus (IBV) which is a single-stranded, positive-sense enveloped RNA virus of the *Coronavirus* family [2]. IBV was first described in the 1930s in the USA [3]. The IBV Massachusetts 41 (M41) strain was subsequently isolated and assigned to the Massachusetts serotype [4, 5], which is frequently used in experimental and clinical research [6, 7]. IBV is liable to mutate and recombine, which gives rise to multiple serotypes [2, 8]. Generally, the available commercial IB vaccines may not trigger powerful immune responses, provide reciprocal protection among different serotypes of IBV infections, and lead to huge economic losses [9]. Therefore, it is necessary to understand the mechanism of host immune responses to IBV infection.

Macrophages are important innate immune effectors against microbial infections and participate in innate immune responses and the subsequent acquired immunity [10]. Pattern recognition receptors (PRRs) in macrophages can recognize the pathogen-associated molecular pattern (PAMP) [11]. Various intracellular signals are triggered subsequently to promote the production of immunomodulation molecules [12, 13] and activate the processing pathways of antigen presentation in macrophages to switch on acquired immune responses [14]. Also, macrophage-programmed cell death-related genes changed in defense against pathogens [6, 15]. It has been shown that IBV could significantly increase the number of macrophages in chicken respiratory tracts [16]. However, the immune function and immune regulation of macrophages in IBV infection remain mostly unclear. In this study, HD11 chicken macrophage cells and chicken peripheral blood mononuclear cell-derived macrophages (PBMCs-Mφ) were infected with IBV M41 strain, and the dynamic changes of macrophage functions and gene expressions of related proteins/mediators in innate and acquired immunity, inflammation, autophagy and apoptosis were systematically analyzed.

## Materials and methods

### Cells and virus

HD11 cells were kindly provided by Dr. Yulong Gao (Harbin Veterinary Research Institute, Chinese Academy of Agricultural Science). HD11 cells were cultured in RPMI 1640 complete medium at 1.5 × 10^5^ cells/mL and were kept at 41 °C in 5% CO_2_. PBMCs-Mφ were separated from chicken blood based on a previous method with minor modifications [17]. In brief, pooled whole blood in 10 U/mL heparins (Ncbiotech Co., Ltd, Harbin, China) was collected from 3-month-old specific-pathogen-free (SPF) chickens by heart punctures. PBMCs were separated by Ficoll-Hypaque (Tianjin Hao Yang Biological Manufacture Co., Ltd, Tianjin, China) density gradient centrifugation according to the manufacturer’s instructions. After being cleaned with 1 × PBS (pH 7.2), PBMCs were seeded in 2 × 10^7^ cells/mL in RPMI 1640 complete medium for 24 h and incubated at 41 °C in 5% CO_2_. Finally, nonadherent cells in the supernatant were removed. The procedures of the experiment complied with the Northeast Agriculture University Health Guidelines for the Care and Use of Laboratory Animals.

IBV strain M41 (Accession number: DQ834384.1) was preserved in the Veterinary Pathology Laboratory, College of Veterinary Medicine in Northeast Agricultural University. The virus was propagated in 9–11-day-old SPF chicken embryos.

### KUL01 + cell assay

The purity of HD11 cells and PBMCs-Mφ were evaluated with flow cytometry via staining of chicken macrophage marker, KUL01 [18]. PBMCs-Mφ and HD11 cells were dispersed with 1% trypsin into a single-cell solution and fixed with 1% paraformaldehyde for 30 min. The cells were incubated with mouse anti-chicken monocyte/macrophage antibodies KUL01 (1: 50, Bio-Rad, USA) for 30 min. After being washed 3 times with PBS, FITC labeled goat anti-mouse IgG (1: 50, ZSGB-Biotechnology Co., Ltd, Beijing, China) was added and incubated in the dark for 30 min. Washed cells were assayed by flow cytometry (BD FACSAria II, USA).

### IBV infection in macrophages

HD11 cells and PBMCs-Mφ were seeded in 6-well plates and grown to 80% confluence. Macrophages were infected with IBV at a multiplicity of infection (MOI) of 10. After being incubated for 2 h at 41 °C, the virus was replaced with RPMI 1640 supplemented with 2% FBS. Cytopathic effects (CPE) were observed daily. Virus titers were determined by endpoint dilutions as 50% tissue culture infective dose (TCID_50_) at 6, 12, 18, 24, 30, 36, 42 and 48 h post-infection (hpi) by the Reed-Muench method, as described previously [19].

Quantitative real-time polymerase chain reaction (qRT-PCR) was also carried out to detect IBV. Total RNA was extracted at 12, 24, 36, 48 hpi using TRIzol reagent (Invitrogen, Shanghai, China) according to the manufacturer’s instructions. Total RNA of 1 μg in each sample was reverse transcribed into cDNA using PrimeScript™ RT reagent Kit with gDNA Eraser (Perfect Real Time) (Takara Biomedical Technology Co., Ltd, Beijing, China). IBV copies were measured by IBV N gene copies using fluorescence quantitative PCR reagent (Bioteke Corporation, Beijing, China). The primers were designed according to the IBV N gene (Accession number: FJ904723.1) and are shown in Table 1. The steps for thermal cycling were as follows: 94 °C, 2 min for denaturation and 40 cycles of PCR (94 °C, 15 s; 60 °C, 30 s).

**Table 1:** Primer sequences for qRT-PCR

Gene	Primer Sequences (5′–3′)	Product length (bp)	Accession number
IBV N	F: CAAGCTAGGTTTAAGCCAGGT	218	FJ904723.1
	R: TCTGAAAACCGTAGCGGATAT		
TLR3	F: ACCCGGATTGCAGTCTCAGTA	95	NM_001011691.3
	R: CACTGTCCTTGCAGGCTGAG		
TLR7	F: ACCGTCGCCTCAAGGAAGTCC	145	NM_001011688.2
	R: ACGCAGTTGCACCTGAAGTCAATC		
MyD88	F: AAGGTGTCGGAGGATGGTGGTC	120	NM_001030962.4
	R: GGAATCAGCCGCTTGAGACGAG		
MDA5	F: TCAGGAGGAGGACGACCACGAT	168	GU570144.1
	R: TTCCCACGACTCTCAATAACAG		
IFN-α	F: GGACATGGCTCCCACACTAC	75	GU119896.1
	R: TCCAGGATGGTGTCGTTGAAG		
IFN-β	F: GCCCACACACTCCAAAACACTG	151	GU119897.1
	R: TTGATGCTGAGGTGAGCGTTG		
IFN-γ	F: CACTGACAAGTCAAAGCCGC	87	NM_205149.1
	R: ACCTTCTTCACGCCATCAGG		
MHCI	F: GCCAACACGGACCAGCAGTAC	81	NM_001097530.1
	R: GTCCAGGTTCTCGCGGTCAATC		
MHCII	F: GTTCTACCAGCGTTCGGAAGGC	101	DQ207939.1
	R: TCTGAGCGGCGTCCAACTCC		
Fc receptor	F: TGTGAGGTGCGGACGGAGAG	195	AM412311.1
	R: TCGGTGCCAGGAGAAGGAGATG		
MARCO	F: CACATAAGCGAGCCTCGAATCCAG	81	NM_204736.1
	R: CAGCAGCAGCAGGTAGATGACAAG		
CD36	F: ACCAGACCAGTAAGACCGTGAAGG	154	NM_001030731.1
	R: ATGTCTAGGACTCCAGCCAGTGTG		
MIF	F: ATTGGCAAGATTGGAGGG	127	M95776.1
	R: CGTTGGCAGCATTTATGTC		
CCL4	F: GCAGTTGTTCTCGCTCTTC	192	NM_204720.1
	R: GCGCTCCTTCTTTGTGAT		
K60	F: GCTGCTGTCATGGCTCTT	278	Y14971.1
	R: TTGGTGTCTGCCTTGTCC		
XCL1	F: ATGAAACTCCACGCCACAGTT	294	NM_205046.1
	R: TTATCTTCTTCTGGTAGTACG		
CXCL12	F: TGTCGGAGGAGAAGCCTGTCAG	158	NM_204510.1
	R: CACTTGCTTGCTGTTGCTCTTGAG		
IL-1β	F: TGGGCATCAAGGGCTACA	244	Y15006.1
	R: TCGGGTTGGTTGGTGATG		
IL-6	F: ATGGTGATAAATCCCGATGAAG	153	NM_204628.1
	R: CCTCACGGTCTTCTCCATAAAC		
TNF-α	F: CAGATGGGAAGGGAATGAAC	268	NM_204267.1
	R: AGAGCATCAACGCAAAAGGG		
NF-κB	F: TCAACGCAGGACCTAAAGACAT	162	M86930.1
	R: GCAGATAGCCAAGTTCAGGATG		
iNOS	F: AGTGGTATGCTCTGCCTGCT	171	NM_204961.1
	R: CCAGTCCCATTCTTCTTCC		
IL-10	F: CAGCACCAGTCATCAGCAGAGC	94	NM_001004414.2
	R: GCAGGTGAAGAAGCGGTGACAG		
PPAR-γ	F: GGGCGATCTTGACAGGAA	175	AB045597.1
	R: GCCTCCACAGAGCGAAAC		
LC3II	F: GTACGAGAGCGAGAAGGACG	83	NM_001031461.1
	R: AGACGGAAGATTGCACTCCG		
mTOR	F: CATGTCAGGCACTGTGTCTATTCTC	77	XM_417614.5
	R: CTTTCGCCCTTGTTTCTTCACT		
Beclin-1	F: TGGCTTTCTTGGACTGTGTG	125	NM_001006332.1
	R: ACCACCACTGCCACCTGTAT		
Caspase-3	F: CCACCGAGATACCGGACTGT	176	NM_204725.1
	R: AACTGCTTCGCTTGCTGTGA		
Bax	F: ACTCTGCTGCTGCTCTCCTCTC	174	XM_025145468.1
	R: ATCCACGCAGTGCCAGATGTAATC		
Bcl-2	F: GAGTTCGGCGGCGTGATGTG	92	NM_205339.2
	R: TTCAGGTACTCGGTCATCCAGGTG		
β-actin	F: ATTGCTGCGCTCGTTGTT	173	K02173.1
	R: CTTTTGCTCTGGGCTTCA		

F: Forward primer for qRT-PCR, R: Reverse primer for qRT-PCR

### Cell viability assay

A CCK-8 reagent (MCE, USA) was used to detect cell viability. UV-IBV was prepared by inactivating IBV M41 with UV germicidal light for 30 min. Cells were seeded into 96-well plates and infected with 10 MOI IBV, or incubated with UV-IBV (same amount of virion to 10 MOI IBV), or PBS (Mock cells) in RPMI 1640 supplemented with 2% FBS. CCK-8 reagent was added to the cells at 12, 24, 36, and 48 hpi, and they were incubated for 2.5 h at 41 °C. The optical density (OD) values were measured with a microplate reader (ELx808, BioTek, USA) at 450 nm, as previously described [20]. Cell viability was calculated according to the formula in the manufacturer’s instructions: Cell viability (%) = [optical density (OD) of Mock or IBV-infected or UV-IBV-treated cells-OD of medium]/[OD of Mock cells-OD of medium] × 100.

### Nitric oxide assay

Cells were seeded into 6-well plates. Supernatants of Mock cells, IBV-infected cells, and UV-IBV-treated cells were collected at 12, 24, 36, and 48 hpi and centrifuged at 1000 *g* for 30 min at 4 °C. Nitric oxide (NO) content in the supernatant was detected by a NO assay kit (Nanjing Jiancheng Bioengineering Institute, Nanjing, China), as previously described [21].

### Macrophage phagocytic ability assay

Phagocytic functions of macrophages were assayed according to the method of Lee et al. [17]. Briefly, yellow-green fluorescent latex beads (50 beads/cell, Sigma, USA) were used to incubate with Mock cells, UV-IBV-treated cells and IBV-infected cells at 24 hpi at 41 °C for 2 h. Cells were then separated by 1% trypsin for macrophage phagocytic ability assay using flow cytometry. At the meantime, cells in these 3 groups were fixed with methanol for 10 min, and stained with 0.01 mg/mL propidium iodide solution for 20 min for observation of phagocytic function under fluorescence microscopy (Nikon, Japan).

### QRT-PCR assay of gene expression

Total RNA of cells was extracted and reverse transcribed into cDNA using PrimeScript™ RT reagent Kit with gDNA Eraser at 12, 24, 36, and 48 hpi. QRT-PCR reagents were used to detect the relative gene expressions of related factors in innate immunity (TLR3, TLR7, MyD88, MDA5, IFN-α, IFN-β, IFN-γ), acquired immunity (MHCI, MHCII, Fc receptor, MARCO, CD36), chemokines (MIF, CCL4, K60, XCL1, CXCL12), inflammation (IL-1β, IL-6, TNF-α, NF-κB, iNOS, IL-10, PPAR-γ), autophagy (LC3II, mTOR, Beclin-1) and apoptosis (Caspase-3, Bax, Bcl-2). The primers are listed in Table 1. β-actin was chosen as a reference gene. The steps for thermal cycling were as follows: 94 °C, 2 min for denaturation and 40 cycles of PCR (94 °C, 15 s; 60 °C, 30 s). The expression fold changes were calculated using the 2^−△△CT^ method [22].

### Statistical analyses

Each treatment was analyzed in triplicate, values were expressed as mean ± SD and statistical significances were assessed by Duncan multiple range test of SPSS 19.0 software using the one-way ANOVA method. Probabilities of *p* < 0.05 and *p* < 0.01 were preset for statistical significance. Flow cytometry analyses were performed on FlowJo software (version 10.0.6). Figures were created with Image J and GraphPad Prism (version 5.0) software.

## Results

### KUL01 + cell percentages of HD11 cells and PBMCs-Mφ

**Figure 1:**
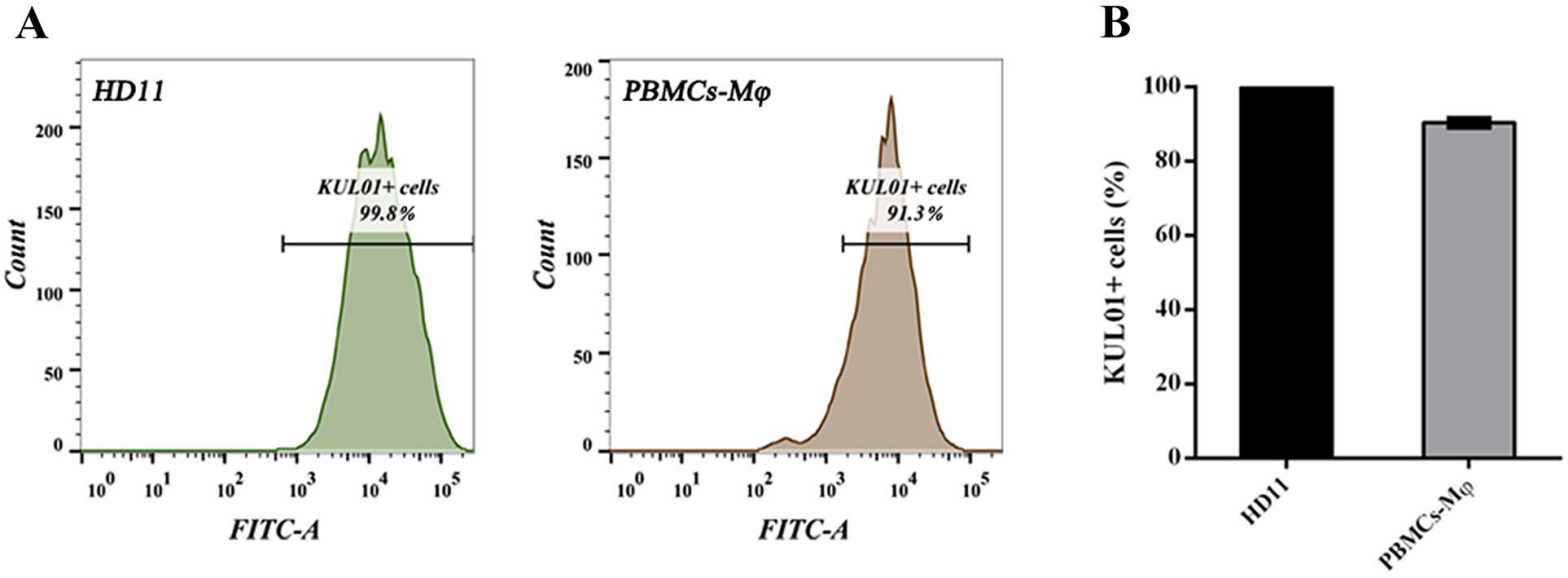
**KUL01 + cell evaluation**. **A** KUL01 + cells in HD11 cells and PBMCs-Mφ were evaluated by flow cytometry. **B** The mean percentages of KUL01 + macrophages in HD11 cells and PBMCs-Mφ are shown in histograms. Data presented as means ± SD (*n* = 3).

Both HD11 cells and PBMCs-Mφ showed high positive fluorescent signals (Figure 1A). The percentages of KUL01 + cells in HD11 cells and PBMCs-Mφ were 99.8 ± 0.1% and 91.3 ± 1.2%, respectively (Figure 1B), indicating these two kinds of macrophage satisfied the needs of the experiments.

### IBV M41 infection in macrophages

**Figure 2:**
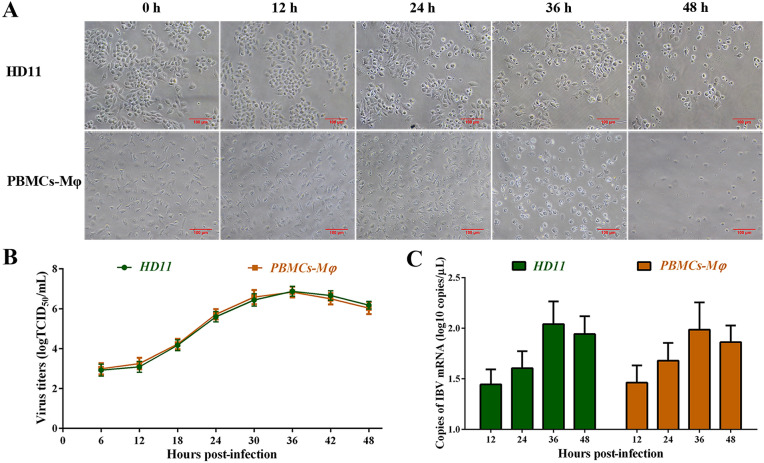
**IBV M41 infection in macrophages.** Macrophages infected with IBV at MOI of 10. **A** CPE observation. CPE results of IBV-infected HD11 cells and PBMCs-Mφ were recorded at 0, 12, 24, 36, 48 hpi. **B** IBV growth curves. Virus titers in IBV-infected HD11 cells and PBMCs-Mφ from 6 to 48 hpi were tested by TCID_50_ method. **C** IBV copies. Virus copies of IBV-infected HD11 cells and PBMCs-Mφ from 12 to 48 hpi were tested by qRT-PCR method. IBV mRNA copies were calculated by the formula: y = − 2.8207x + 40.827 (R^2^ = 0.991). Data presented as means ± SD (*n* = 3).

HD11 cells and PBMCs-Mφ were infected with IBV at an MOI of 10. CPE, qRT-PCR and virus titer assays were carried out. After 5 times of adaptive cell culture of IBV M41 strain in both kinds of cell, the typical CPE appeared in macrophages at 24 hpi, and peaked at 36 hpi (Figure 2A). QRT-PCR and TCID_50_ results showed IBV replication increased in a time-dependent manner, while there was a decrease at 48 hpi (Figures 2B, C). The results indicate IBV M41 could infect HD11 cells and PBMCs-Mφ.

### IBV M41 decreased macrophage viability

**Figure 3:**
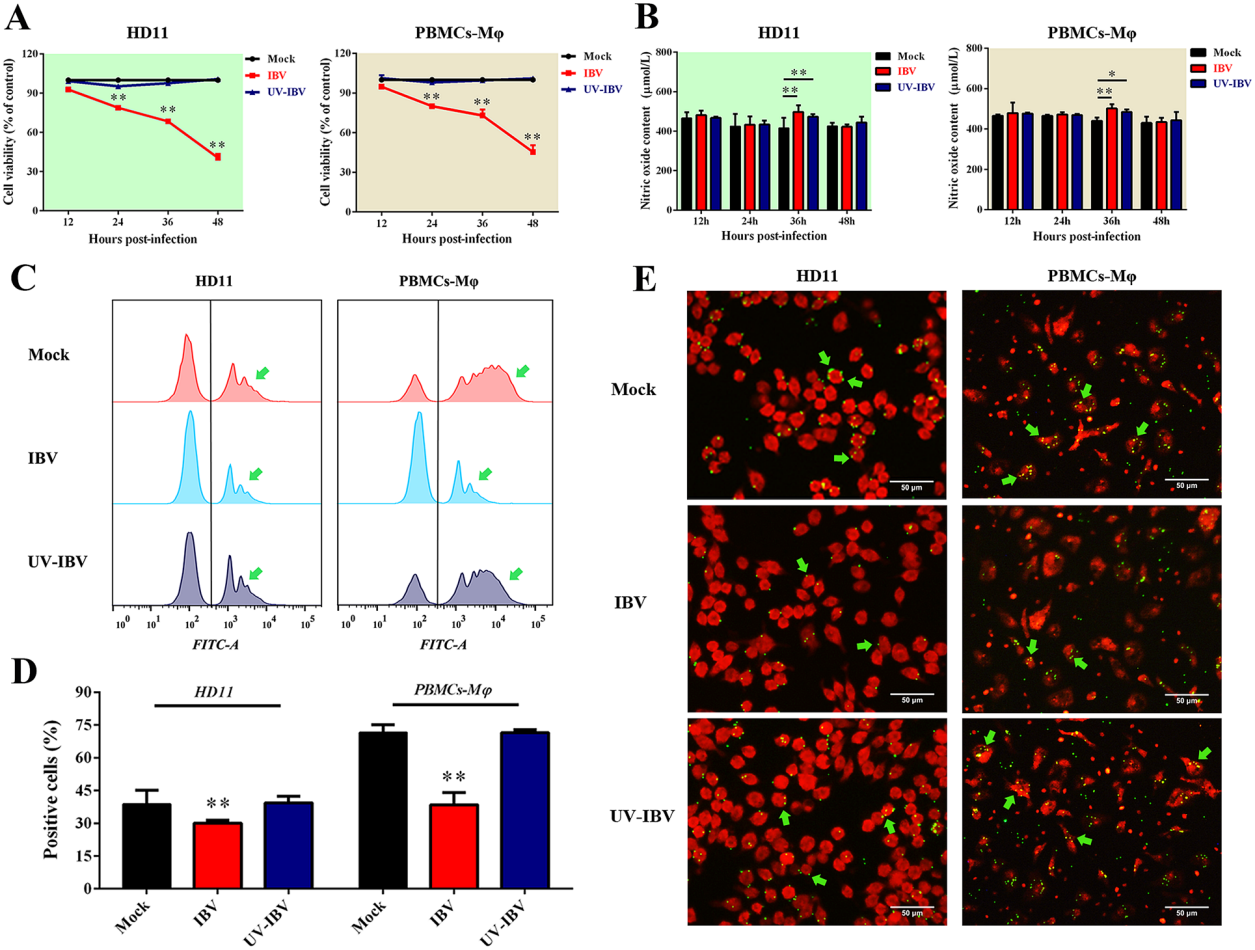
**Macrophages activity assays.** **A** CCK-8 assay. CCK-8 was used to detect the viability of HD11 cells and PBMCs-Mφ infected with IBV or treated with UV-IBV or PBS (Mock cells) from 12 to 48 hpi. **B** NO content assay. NO content of culture supernatants of IBV-infected, UV-IBV-treated or Mock cells were measured from 12 to 48 hpi. **C**, **D** Macrophage phagocytic function assay by flow cytometry. Phagocytic functions of chicken macrophages were detected at 24 hpi by phagocytosis of yellow-green fluorescent beads. Flow cytometry histogram shows fluorescent intensity of cells containing yellow-green fluorescent beads (green arrow) and cells not containing yellow-green fluorescent beads in three groups (**C**). The mean percentages of positive cells post-infection are shown in histograms (**D**). Data presented as means ± SD (*n* = 3). **E** Macrophage phagocytic function observation under fluorescent microscopy. Beads (green arrow) and nucleus (red) were observed under the inverted fluorescence microscope. Data presented as mean ± SD (*n* = 3). * means the significance of between IBV-infected or UV-IBV-treated cells with Mock cells. * means *p* < 0.05, ** means *p* < 0.01.

Cell viability was tested. CCK-8 assay results showed that cell viability of IBV-infected macrophages decreased in a time-dependent manner (*p* < 0.01), while there were no significant changes in Mock cells and UV-IBV-treated cells (Figure 3A).

### IBV M41 activated macrophage pathogen elimination function

NO content was examined to determine the pathogen elimination function of macrophages. NO secreted by macrophages is a key aspect of the antimicrobial response [23]. NO content increased significantly in IBV-infected (HD11/PBMCs-Mφ: *p* < 0.01) and UV-IBV-treated (HD11: *p* < 0.01, PBMCs-Mφ: *p* < 0.05) cell supernatants at 36 hpi compared with Mock cells (Figure 3B). The up-regulated NO content of UV-IBV-treated cells was lower than that of IBV-infected cells, indicating that IBV replication stimulates NO production in chicken macrophages.

### IBV M41 affected macrophage phagocytic function

Phagocytic function of macrophages was determined by detecting the percentage of macrophages phagocytizing yellow-green fluorescent latex beads by flow cytometry and fluorescence microscopy. Flow cytometry results showed less yellow-green fluorescence in IBV-infected cells compared with Mock cells at 24 hpi, HD11 cells (Mock: 38.8%, IBV: 30.0%; *p* < 0.01) and PBMCs-Mφ (Mock: 71.5%, IBV: 38.4%; *p* < 0.01). There were no significant changes in UV-IBV-treated cells (Figure 3C, D). Fluorescence microscopy results were consistent with those of the flow cytometry assays (Figure 3E). These indicate that IBV infection could significantly damage the phagocytic functions of macrophages.

### IBV M41 activated macrophage innate immunity

**Figure 4:**
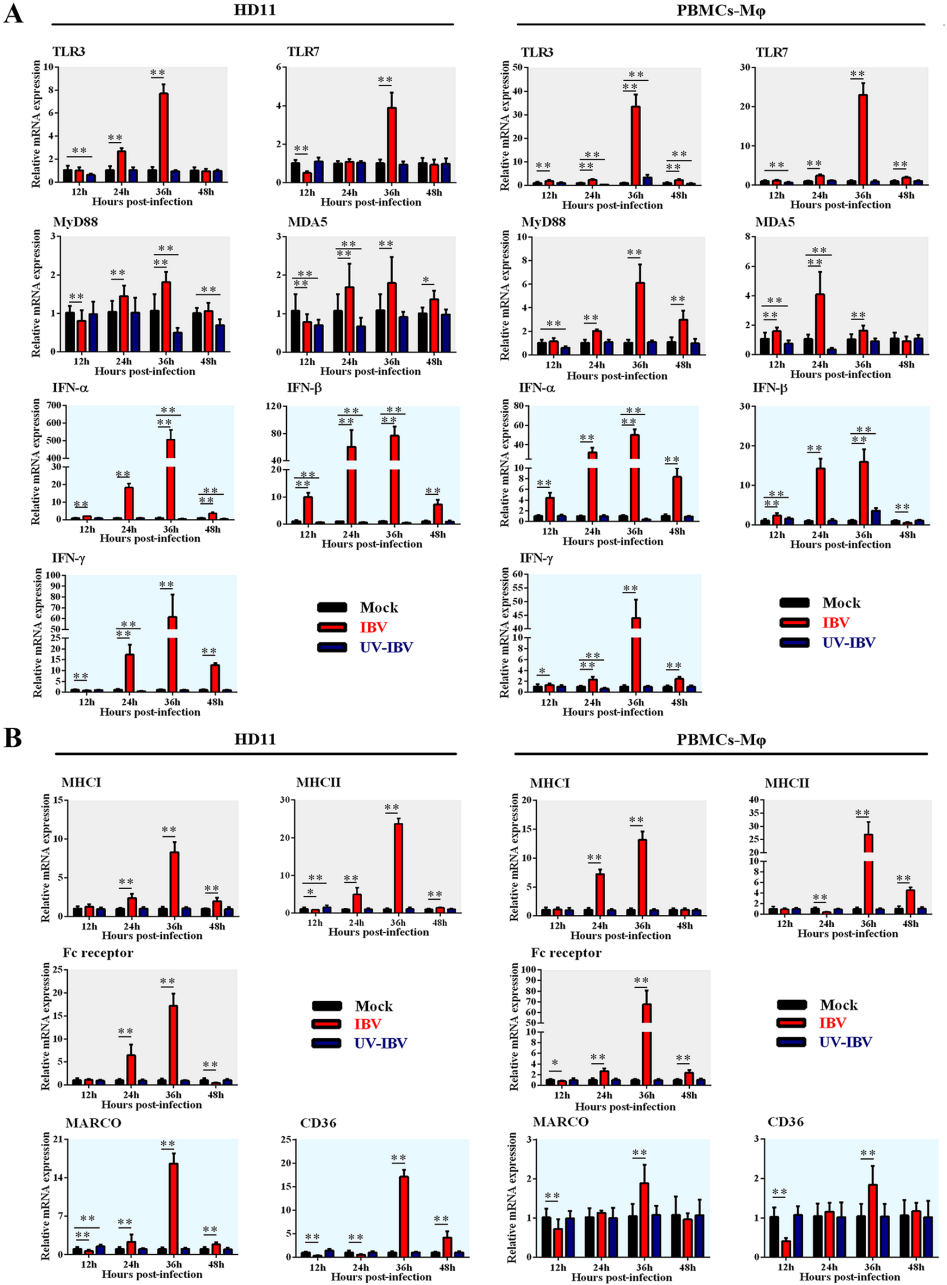
**Antiviral innate and acquired immunity-related gene expressions.** **A** Antiviral innate immunity-related factors mRNA expressions. Relative mRNA expressions of TLRs (TLR3, TLR7), MyD88, MDA5, IFNs (IFN-α, IFN-β, IFN-γ) in IBV-infected, UV-IBV-treated cells and Mock cells were detected by qRT-PCR method. β-actin acted as a reference gene. **B** Acquired immunity-related gene mRNA expressions. Relative mRNA expressions of MHC molecules (MHCI, MHCII), Fc receptor, MARCO, CD36 in IBV-infected, UV-IBV-treated cells and Mock cells were detected by qRT-PCR method. β-actin acted as a reference gene. Data presented as mean ± SD (*n* = 3). * means the significance of between IBV-infected or UV-IBV-treated cells with Mock cells. * means *p* < 0.05, ** means *p* < 0.01.

Toll-like receptors (TLRs) are PRRs that detect characteristic microbial motifs to signal the presence of invading microbial organisms [24]. Among them, TLR3 and TLR7 recognize viral or non-viral RNA [25]. Myeloid differentiation factor 88 (MyD88) is a key linker molecule in the TLRs signaling pathway [26]. As is known, TLR3/7-MyD88 are classic pathways involved in host recognition of viruses and innate immunity. In IBV-infected HD11 cells, the gene expressions of TLR7 and MyD88 both decreased at 12 hpi (*p* < 0.01). As the virus replicated, the gene expressions of TLR3/7 and MyD88 showed obvious upward trends (*p* < 0.01) and peaked at 36 hpi. The general expression trends of TLR3/7 and MyD88 in PBMCs-Mφ were consistent with those of HD11 cells. In UV-IBV-treated HD11 cells, the gene expression of TLR3 decreased significantly at 12 hpi (*p* < 0.01), and MyD88 at 36 and 48 hpi (*p* < 0.01). In UV-IBV-treated PBMCs-Mφ, TLR7 and MyD88 gene expressions decreased at 12 hpi (*p* < 0.01). TLR3 expression decreased at 24 and 48 hpi (*p* < 0.01), and increased at 36 hpi (*p* < 0.01). However, the TLR3 up-regulation in UV-IBV-treated PBMCs-Mφ was lower than that of IBV-infected cells (Figure 4A). These results indicate that viral replication is involved in IBV-induced TLR3/7-MyD88 modulation. By comparing the increasing mRNA expressions of TLR3 (HD11: sevenfold, PBMCs-Mφ: 33-fold) and TLR7 (HD11: fourfold, PBMCs-Mφ: 22-fold) at 36 hpi in IBV-infected cells, it can be concluded that IBV infection activates the TLR3/7 signaling pathways, especially the TLR3 signaling pathway.

Cytoplasmic retinoic acid-inducible gene I (RIG-I)-like receptors (RLRs) identify viral RNAs and initiate innate immune responses [27]. Chickens do not have RIG-I [28]. Therefore, the other RLR member, melanoma differentiation associated protein 5 (MDA5), plays an important role in recognizing intracellular viruses in chickens [29]. In IBV-infected HD11 cells, MDA5 expression decreased at 12 hpi (*p* < 0.01), then increased at 24, 36 and 48 hpi (24 and 36 hpi: *p* < 0.01, 48 hpi: *p* < 0.05). The trend of MDA5 expression in PBMCs-Mφ was consistent with that of HD11 cells, which increased at 12, 24 and 36 hpi (*p* < 0.01), and peaked at 24 hpi. In UV-IBV-treated HD11 cells and PBMCs-Mφ, the gene expression of MDA5 decreased at 12 and 24 hpi (*p* < 0.01, Figure 4A).

These results suggest that IBV activates antiviral innate immunity pathways in macrophages. However, immunosuppression might occur at the early stage of IBV infection.

### IBV M41 upregulated IFN expressions in macrophages

IFNs, including type I (IFN-α and IFN-β) and type II (IFN-γ), play important roles in antiviral defense, inhibit viral replication and work as immune effectors/modulators [30, 31]. In IBV-infected HD11 cells, the expressions of IFN-α and IFN-β increased at 12 hpi (*p* < 0.01), while the expression of IFN-γ decreased (*p* < 0.01). As the virus replicated, IFNs expressions showed upward trends, increasing at 24, 36, and 48 hpi (*p* < 0.01), and peaking at 36 hpi. The IFNs expression trends of PBMCs-Mφ were consistent with those of HD11 cells. In UV-IBV-treated HD11 cells, the expression of IFN-α decreased at 36 and 48 hpi (*p* < 0.01), IFN-β decreased at 12, 24 and 36 hpi (*p* < 0.01), and IFN-γ decreased at 24 hpi (*p* < 0.01). In UV-IBV-treated PBMCs-Mφ, IFN-α and IFN-γ expressions decreased at 36 and 24 hpi, respectively (*p* < 0.01). However, IFN-β expression increased at 36 hpi (*p* < 0.01, Figure 4A).

These results indicate that IBV could activate IFNs production in macrophages, which has a relationship with viral replication. Comparing the increasing expressions of IFNs, IFN-α up-regulations (HD11: 490-fold, PBMCs-Mφ: 49-fold) were the highest.

### IBV M41 activated macrophage acquired immunity

Major histocompatibility complex (MHC) is a family of cell surface molecules, including MHC class I and II, which is involved in antigen presentation and activation of T cells [32]. Fc receptor is involved in receptor-mediated antibody-dependent internalization of immune complexes destined for intracellular degradation [33]. In IBV-infected HD11 cells, MHCII expression decreased at 12 hpi (*p* < 0.05). As the virus replicated, elevated expressions of MHC molecules and Fc receptors appeared at 24 and 36 hpi (*p* < 0.01), and peaked at 36 hpi. The expression trends of MHC molecules and Fc receptor in PBMC-Mφ were consistent with those of HD11 cells. In IBV-infected PBMCs-Mφ, MHC molecules and Fc receptor expressions were up-regulated, but MHCII expression decreased at 24 hpi (*p* < 0.01). In the UV-IBV-treated HD11 cells, only MHCII expression increased at 12 hpi (*p* < 0.01, Figure 4B).

Scavenger receptors and mannose receptors, parts of PRRs, are also involved in pathogen elimination. CD36 is a scavenger receptor involved in immunity, metabolism and angiogenesis [34]. Mannose receptor, MARCO, plays a role in pathogen clearance and inflammatory ligand recognition [35]. In IBV-infected HD11 cells, the expressions of MARCO decreased at 12 hpi significantly (*p* < 0.01), and CD36 at 12, and 24 hpi (*p* < 0.01). Increased expressions of both genes appeared and peaked at 36 hpi (*p* < 0.01). The expression trends of MARCO and CD36 in PBMC-Mφ were similar to those in HD11 cells. Both MARCO and CD36 expressions down-regulated at 12 hpi (*p* < 0.01), and then increased and peaked at 36 hpi (*p* < 0.01). For the UV-IBV-treated HD11 cells, there were no distinct changes in the two kinds of cell (Figure 4B).

These results indicate that IBV inhibits the ability of macrophages to eliminate pathogen and antigen presentation at the early stage of viral infection, but do not affect the latter activation of acquired immunity. Among these genes, the expression of MHCII was the highest (23-fold) compared with other genes in IBV-infected HD11 cells, and MHCII (26-fold) and Fc receptor (67-fold) in IBV-infected PBMCs-Mφ, showing that IBV infection mainly activates MHCII and Fc receptors.

### IBV M41 activated macrophage chemokine expressions

**Figure 5:**
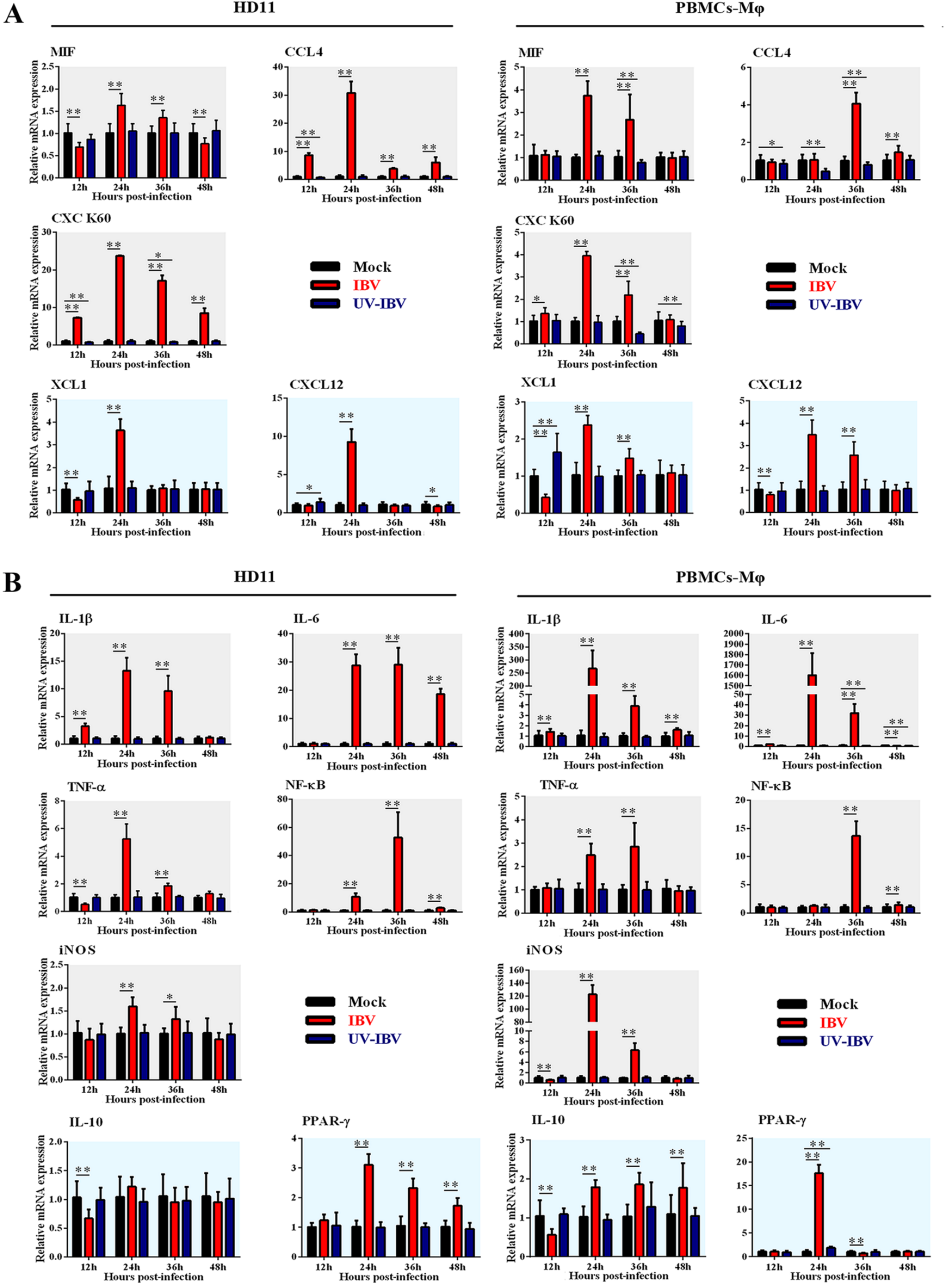
**Chemokines and inflammatory factors gene expressions.** **A** Chemokine mRNA expressions. Relative mRNA expressions of MIF, CCL4, CXC K60, XCL-1, CXCL12 in IBV-infected, UV-IBV-treated cells and Mock cells were detected by qRT-PCR method. β-actin acted as a reference gene. **B** Inflammatory factor mRNA expressions. Relative mRNA expressions of IL-1β, IL-6, NF-κB, TNF-α, iNOS, IL-10 and PPAR-γ in IBV-infected, UV-IBV-treated cells and Mock cells were detected by qRT-PCR method. β-actin acted as a reference gene. Data presented as mean ± SD (*n* = 3). * means the significance of between IBV-infected or UV-IBV-treated cells with Mock cells. * means *p* < 0.05, ** means *p* < 0.01.

Chemokines are involved in host immune responses and inflammatory processes [36, 37]. Macrophage migration inhibitory factor (MIF), CCL4 (also known as macrophage inflammatory protein-1β) and CXC K60, that can activate macrophages and T cells, are involved in cell migration, and promote cell maturation [38–40]. In IBV-infected HD11 cells, the gene expressions of MIF, CCL4 and CXC K60 up-regulated significantly (*p* < 0.01), and peaked at 24 hpi, but there was a marked decrease of MIF expression at 12 hpi (*p* < 0.01). The expression trends in PBMCs-Mφ were similar to those in HD11 cells. In UV-IBV-treated HD11 cells, CCL4 expression decreased at 12 hpi (*p* < 0.01) and CXC K60 decreased at 12 (*p* < 0.01) and 36 hpi (*p* < 0.05). In UV-IBV-treated PBMCs-Mφ, the decreased expression of MIF showed at 36 hpi (*p* < 0.01), CCL4 decreased at 24 and 36 hpi (*p* < 0.01), and CXCK60 decreased at 36 hpi (*p* < 0.01, Figure 5A).

XCL1 (also known as lymphotactin) and CXCL12 (also known as stromal cell derived factor-1) have a strong chemotactic effect on lymphocytes, involved in inflammatory responses [41, 42]. In IBV-infected HD11 cells, there was an abrupt increase in XCL1 and CXCL12 expressions at 24 hpi (*p* < 0.01). However, XCL1 and CXCL12 expressions decreased significantly at 12 and 48 hpi, respectively (*p* < 0.01). The XCL1 and CXCL12 expression trends in PBMCs-Mφ were similar to those in HD11 cells (*p* < 0.01), but there was a gradual decrease of XCL1 and CXCL12 expressions at 36 hpi (*p* < 0.01). In the UV-IBV-treatment experiment, there were only increased expressions of CXCL12 in HD11 cells (*p* < 0.05) and of XCL1 in PBMCs-Mφ at 12 hpi (*p* < 0.01, Figure 5A).

These results suggest that IBV could activate the chemotaxis in macrophages with temporary suppression at the early stage of infection. The mRNA expressions of CCL4 (29-fold) and MIF (24-fold) were the highest in IBV-infected HD11 cells compared with other chemokines, indicating that IBV-infected macrophages mainly secrete CCL4 and MIF.

### IBV M41 up-regulated inflammatory factor expressions in macrophages

Interleukin (IL)-1β, IL-6 and tumor necrosis factor (TNF)-α are classic pro-inflammatory cytokines and activate a variety of stress responses [43–45]. Transcription factor Nuclear factor-κB (NF-κB) is a redox-sensitive transcription factor and modulates ILs production [46]. Inducible nitric oxide synthase (iNOS) is involved in NO synthesis and the pro-inflammatory responses of macrophages [47]. IL-10 is an anti-inflammatory cytokine which controls antigen presentation function and the ability to synthesize cytokines [48]. Peroxisome proliferator-activated receptor (PPAR)-γ is related to inflammation inhibition [49, 50]. In IBV-infected HD11 cells, pro-inflammatory cytokines showed upward trends from 12 to 24 hpi. IL-1β, IL-6, TNF-α, iNOS peaked at 24 hpi (*p* < 0.01), and NF-κB peaked at 36 hpi (*p* < 0.01). The expression trends in PBMCs-Mφ were similar to those in HD11 cells. For anti-inflammatory cytokines, IL-10 decreased at 12 hpi in IBV-infected HD11 cells (*p* < 0.01), and PPAR-γ was up-regulated at 24, 36, and 48 hpi (*p* < 0.01). However, in PBMCs-Mφ, there was an obvious increase in IL-10 expression at 24, 36, and 48 hpi (*p* < 0.01), and in PPAR-γ at 24 hpi (*p* < 0.01), respectively (Figure 5B).

These results indicate that IBV induces macrophage pro-inflammatory and anti-inflammatory responses. In IBV infection, there were increased expressions of IL-6 mRNA (27-fold) in HD11 cells. The relative mRNA expression of IL-1β (246-fold), IL-6 (1560-fold), iNOS mRNA (118-fold) in PBMCs-Mφ had higher expressions compared with other cytokines.

### IBV M41 induced autophagy in macrophages

**Figure 6:**
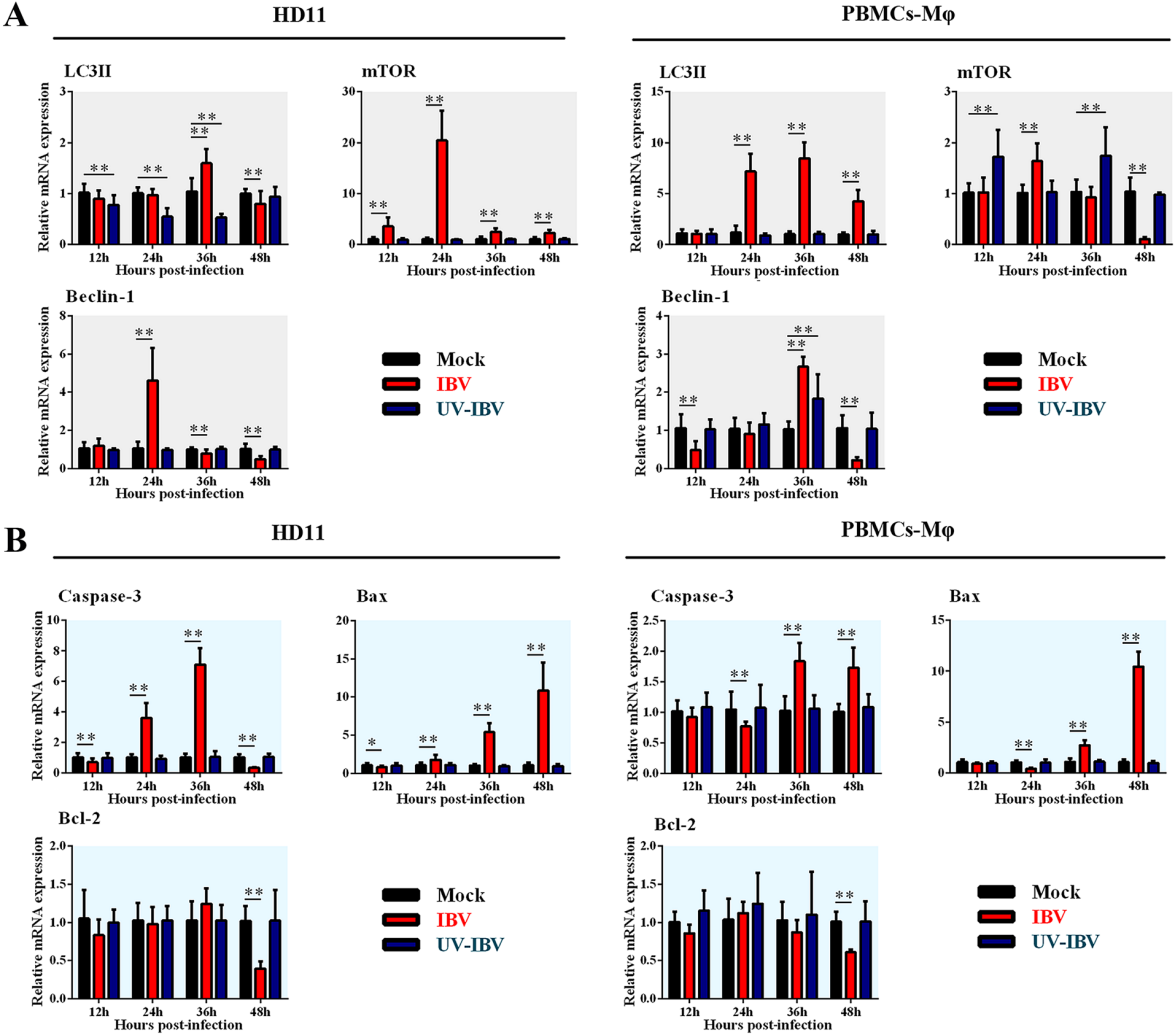
**Gene expressions of related proteins in apoptosis and autophagy.** **A** Autophagy-related gene mRNA expressions. Relative mRNA expressions of LC3II, mTOR and Beclin-1 in IBV-infected, UV-IBV-treated cells and Mock cells were detected by qRT-PCR method. β-actin acted as a reference gene. **B** Apoptosis-related gene mRNA expressions. Relative mRNA expressions of Caspase-3, Bax, Bcl-2 in IBV-infected, UV-IBV-treated cells and Mock cells were detected by qRT-PCR method. β-actin acted as a reference gene. Data presented as mean ± SD (*n* = 3). * means the significance of between IBV-infected or UV-IBV-treated cells with Mock cells. * means *p* < 0.05, ** means *p* < 0.01.

Microtubule-associated protein 1 light chain 3 II (LC3II) is an autophagic target protein involved in the formation of autophagosomes [51]. Mammalian target of rapamycin (mTOR) and Beclin-1 are key proteins involved in the autophagic pathways [52, 53]. LC3II expression peaked at 36 hpi in IBV-infected HD11 cells and PBMCs-Mφ (*p* < 0.01). mTOR and Beclin-1 increased to a peak at 24 or 36 hpi (*p* < 0.01), and subsequently decreased rapidly in IBV-infected HD11 cells and PBMCs-Mφ, respectively (Figure 6A). This indicates that IBV infection could induce autophagy at the middle term of viral replication and participate in the disease pathogenic process.

### IBV M41 induced apoptosis in macrophages

Caspase-3 is an executive factor of apoptosis [54]. Bcl-2-associated X (Bax) is a classic pro-apoptotic factor [55]. Bcl-2 is an inhibitor of apoptosis [56]. Caspase-3 expression peaked at 36 hpi in IBV-infected HD11 cells and PBMCs-Mφ (*p* < 0.01). However, Caspase-3 expression was down-regulated at 12 and 48 hpi in IBV-infected HD11 cells (*p* < 0.01), and at 24 hpi in PBMCs-Mφ (*p* < 0.01), respectively. In IBV-infected HD11 cells, the expression of Bax decreased at 12 hpi (*p* < 0.05), and 24 hpi in PBMCs-Mφ (*p* < 0.01). As the virus replicated, the expression of Bax showed an increase trend, and peaked at 48 hpi (*p* < 0.01). Remarkably, decreased expressions of Bcl-2 appeared in both HD11 cells and PBMCs-Mφ at 48 hpi (*p* < 0.01). UV-IBV treatment did not change the mRNA expressions of Caspase-3, Bax and Bcl-2 in macrophages (Figure 6B). These results indicate that viral replication provokes apoptosis in macrophages at the late stage of infection.

## Discussion

Macrophages are an important part of innate immunity [57]. Macrophages respond to external stimuli with rapid changes in their expressions of various related genes. In this study, we established an experimental model of IBV infection of macrophages. The high-purity HD11 cells (99.8%) and PBMCs-Mφ (91.3%) could exhibit typical CPE at 36 h post-IBV M41 strain infection, and the virus could replicate on them. These results demonstrated that both kinds of cell were suitable for studying the interaction of IBV and macrophages and the underlying mechanisms. Macrophage viability and abilities were checked. IBV could affect macrophage viability and damage their phagocytic functions, which are related to viral replication. Han et al. [58] found that IBV Beaudette strain could propagate stably in HD11 cells and the virus titer reached the high level of 10^6.85^ TCID_50_/mL, typical CPE appeared at 36 hpi, and cell viability decreased obviously at the same time. In other words, IBV could impair the viability of chicken macrophages and their biological functions, which was consistent with our results.

Host immune response is divided into innate immunity and acquired immunity [59]. TLRs and RLRs are two types of PRR in the innate immune system [60]. TLR3 and TLR7 are both involved in IBV infection, which is consistent with previous studies [16, 61], and TLR3 may play a key role in IBV-induced viral immune mechanisms. Furthermore, IBV infection activates MDA5 expression [16, 62]. The expressions of the key antiviral molecules, IFNs and other acquired immunity-related genes, which are involved in antigen presentation and pathogen clearance, increased significantly with virus replication. This indicates that IBV activates the innate immune response of macrophages. Interestingly, the gene expressions of certain signal proteins, such as TLR7, MyD88, MDA5, MHCII, and Fc receptor, decreased in HD11 cells and PBMCs-Mφ at 12 hpi, respectively, indicating that IBV could inhibit the immune regulatory function of macrophages at the early stage of its infection.

Macrophages secrete chemokines post-IBV infection, which attract immune competent cells to the sites of infection and inflammation [63]. Meanwhile, cytokines are activated to participate in information transmission, immune regulation and effector functions [43, 44]. Similar to other research, we found that IBV activated the gene expressions of most chemokines and inflammatory cytokines [16, 61, 64], demonstrating a promotion of inflammatory response. Among them, CCL4, IL-1β, IL-6, and iNOS mainly activated and participated in innate immunity of IBV infection. The expressions of MIF, XCL1 and CXCL12 decreased at the early stage of infection, indicating IBV affects macrophage chemotaxis. Moreover, the up-regulation of inflammatory cytokines in IBV infection was higher in percentage terms than in anti-inflammatory cytokines at 12 and 24 hpi, indicating that a pro-inflammatory response is activated at the early stage of IBV infection. From 24 to 48 hpi, the expressions of cytokines gradually decreased, indicating that at the late stage of IBV infection, an anti-inflammatory response is dominant in the macrophages.

Finally, the gene expressions of related proteins involved in apoptosis and autophagy were investigated. There have been reports that IBV could induce autophagy and apoptosis in both chicken and mammalian cells [58, 65, 66]. In this study, there occurred autophagy and apoptosis in HD11 cells and PBMCs-Mφ post-IBV infection. In addition, we found that autophagy appeared and peaked at the period of extensive viral replication, and apoptosis occurred obviously at the later stage of stable virus replication. With virus replication in macrophages, there appeared autophagy firstly and apoptosis subsequently. Furthermore, the gene expression levels of related proteins enhanced post-IBV infection, while there were no obvious changes post-UV-IBV treatment. This further supports the fact that the up-regulation of gene expressions has a close relationship with virus replication.

In conclusion, IBV decreased macrophage phagocytic functions and viability, but strengthened pathogen elimination functions. IBV promoted nearly all the gene expressions of related proteins in macrophages—except some degree of suppression at the earlier stage—to exert its biofunctions in multiple host responses, and the dynamic changes of gene expression had a close relationship with virus replication. This might provide some insight into understanding the immunopathogenesis mechanism of IBV infection.

## Data Availability

The datasets used and/or analysed during the current study are available from the corresponding author upon reasonable request.

## Abbreviations

IBV: Infectious bronchitis virus
UV-IBV: UV-inactivated IBV
PAMP: Pathogen-associated molecular pattern
PRRs: Pattern recognition receptors
PBMCs-Mφ: Peripheral blood mononuclear cells-derived macrophages
SPF: Specific-pathogen-free
MOI: Multiplicity of infection
CPE: Cytopathic effects
TCID_50_: 50% Tissue culture infective dose
NO: Nitric oxide
qRT-PCR: Quantitative real-time polymerase chain reaction
hpi: Hours post-infection
MHC: Major histocompatibility complex
TLRs: Toll-like receptors
MyD88: Myeloid differentiation factor 88
MDA5: Melanoma differentiation associated protein 5
IFNs: Interferons
MIF: Macrophage migration inhibitory factor
iNOS: Inducible nitric oxide synthase
IL: Interleukin
NF-κB: Nuclear factor κB
TNF-α: Tumor necrosis factor-α
PPAR-γ: Proliferator-activated receptor γ
mTOR: Mammalian target of rapamycin
LC3II: Microtubules associated protein 1 light chain 3 II
Bax: Bcl-2-associated X
